# Microparticles Based on Chitosan/Xanthan Gum Polyelectrolyte Complex Modulate the Anti-Inflammatory and Antinociceptive Effects of Ibuprofen and Escin

**DOI:** 10.3390/md24070225

**Published:** 2026-06-26

**Authors:** Ana Ćirić, Nikola Martić, Milana Bosanac, Bojana Andrejić Višnjić, Aleksandar Rašković, Ljiljana Đekić

**Affiliations:** 1 Department of Pharmaceutical Technology and Cosmetology, Faculty of Pharmacy, University of Belgrade, Vojvode Stepe 450, 11221 Belgrade, Serbia; ljiljana.djekic@pharmacy.bg.ac.rs; 2 Department of Pharmacology, Toxicology, and Clinical Pharmacology, Faculty of Medicine, University of Novi Sad, Hajduk Veljkova 3, 21000 Novi Sad, Serbia; nikola.martic@mf.uns.ac.rs (N.M.); aleksandar.raskovic@mf.uns.ac.rs (A.R.); 3 Department of Histology and Embryology, Faculty of Medicine, University of Novi Sad, Hajduk Veljkova 3, 21000 Novi Sad, Serbia; milana.bosanac@mf.uns.ac.rs (M.B.); bojana.andrejic-visnjic@mf.uns.ac.rs (B.A.V.)

**Keywords:** chitosan, xanthan gum, polyelectrolyte complexes, escin, ibuprofen, microparticles, oral drug delivery, anti-inflammatory activity, antinociceptive activity

## Abstract

Polyelectrolyte complex (PEC)-based carriers offer a promising strategy to improve the oral delivery of anti-inflammatory agents with limited bioavailability or variable pharmacodynamic profiles. This study evaluated the anti-inflammatory and antinociceptive effects of previously optimized formulations of chitosan/xanthan gum PEC microparticles loaded with either ibuprofen or escin, using the carrageenan-induced paw edema model, histopathological and cyclooxygenase-2 (COX-2) immunohistochemical analyses, and the hot plate test. Ibuprofen-loaded microparticles significantly reduced paw swelling during the peak inflammatory phase (5–6 h after treatment administration), although no significant differences in overall edema response or antinociceptive activity were observed compared with free ibuprofen. In contrast, escin-loaded microparticles at 10 mg/kg produced the most pronounced anti-inflammatory effect, significantly reducing paw swelling, edema area under the curve (AUC), histopathological lesion scores, and COX-2 expression compared with both the negative control and the corresponding free escin formulation. Escin-loaded microparticles also showed stronger and more sustained antinociceptive activity than free escin. However, the 20 mg/kg formulation did not provide additional anti-inflammatory or antinociceptive benefits. These findings demonstrate that chitosan/xanthan gum PEC microparticles can enhance the pharmacodynamic performance of orally administered anti-inflammatory agents. The magnitude of this effect depended on the incorporated drug and was particularly notable for escin, for which microencapsulation improved both anti-inflammatory and antinociceptive efficacy.

## 1. Introduction

Inflammation is a protective biological response to harmful stimuli. However, when excessive or prolonged, it can lead to chronic inflammatory disorders and tissue damage [1,2,3]. Acute inflammation is often associated with edema and pain, which arise from the action of inflammatory mediators and activation of nociceptive pathways [1,4]. Conventional treatment of inflammatory conditions and associated pain mainly relies on nonsteroidal anti-inflammatory drugs (NSAIDs), corticosteroids, and analgesics such as opioids [5,6]. However, long-term use of these agents is frequently associated with adverse effects, including gastrointestinal irritation, cardiovascular complications, immunosuppression, endocrine disturbances, tolerance, and dependence [7,8,9]. Therefore, developing more effective and safer therapeutic strategies that improve efficacy while reducing systemic adverse effects remains an important pharmaceutical challenge.

Ibuprofen is one of the most widely used NSAIDs and exerts its therapeutic effect primarily through reversible inhibition of COX-2, resulting in reduced synthesis of prostaglandins involved in inflammation, edema, fever, and pain. However, its short elimination half-life, gastrointestinal irritation, and the need for repeated dosing may limit its long-term therapeutic use [10]. To address these limitations, various formulation strategies, including amorphous solid dispersions [11,12,13], lipid-based carriers [14,15,16], and polymeric nanoparticles [17,18], have been investigated to improve ibuprofen dissolution, bioavailability, and therapeutic performance.

Escin, a natural mixture of triterpenoid saponins isolated from horse chestnut (*Aesculus hippocastanum* L.) seeds, has attracted increasing attention as a promising anti-inflammatory and anti-edematous agent. The major pharmacologically active constituent is β-escin, which is considered primarily responsible for its therapeutic effects [19,20,21]. Previous research has shown that escin possesses anti-edematous, anti-inflammatory, venotonic, antioxidant, and vasoprotective properties [22], and increasing evidence suggests its antihyperalgesic potential [23,24]. Unlike ibuprofen, the exact mechanism of action of escin remains incompletely understood and is thought to involve multiple pathways related to inflammation, vascular permeability, and pain modulation [25,26,27,28]. This complex pharmacological profile makes escin particularly attractive for formulation-based pharmacodynamic evaluation.

Despite growing pharmacological interest in escin, studies on advanced carrier systems for its oral delivery remain limited, particularly those designed to enhance its bioavailability and pharmacodynamic effects. Most available research primarily investigates the pharmacological activity of free escin, while formulation strategies to improve its oral therapeutic performance are considerably less explored. Recent work on escin-loaded gelatin nanoparticles has demonstrated enhanced biological efficacy compared with free escin, further supporting the potential of carrier-based systems to improve its therapeutic response [29]. Nevertheless, data on oral polymer-based delivery systems for escin, especially those intended to improve anti-inflammatory and antinociceptive outcomes, remain scarce.

Among advanced oral delivery systems, PECs are a particularly attractive approach due to their simple preparation under mild aqueous conditions, high biocompatibility, pH-responsive drug release, mucoadhesion, and ability to protect drugs from gastric degradation while improving intestinal absorption [30]. Chitosan, obtained by deacetylation of chitin derived predominantly from marine crustacean shells, is one of the most extensively investigated marine biopolymers for pharmaceutical and drug delivery applications. Owing to its biocompatibility, biodegradability, mucoadhesive properties, and ability to form PECs with oppositely charged polymers, chitosan has attracted considerable interest as a marine-derived excipient for oral delivery systems [31]. For ibuprofen, previous studies have shown that chitosan-based PEC systems, such as chitosan/alginate-coated nanofibers and chitosan–synthetic methacrylate copolymer nanoparticles, enable pH-dependent release, prolonged drug release, improved bioavailability, and longer pharmacological activity, confirming their potential for oral drug delivery [32,33].

In our previous studies, chitosan/xanthan gum PEC-based microparticles loaded with ibuprofen and escin were successfully developed and optimized. The selected formulations showed favorable physicochemical properties, pH-dependent release behavior, improved pharmacokinetic performance after oral administration, and a favorable safety profile [34,35,36,37]. However, although these findings indicate significant formulation-related advantages, it remains unclear whether these improvements result in enhanced pharmacodynamic efficacy in vivo.

Therefore, the aim of the present study was to conduct a comprehensive pharmacodynamic evaluation of previously optimized formulations of chitosan/xanthan gum PEC microparticles loaded with ibuprofen and escin. Anti-inflammatory and antinociceptive effects were assessed using carrageenan-induced paw edema, histological and COX-2 immunohistochemical analyses, and the hot plate test. Particular attention was given to determining whether microencapsulation provides different pharmacodynamic benefits for escin and ibuprofen after oral administration.

## 2. Results

### 2.1. Anti-Inflammatory Activity

#### 2.1.1. Anti-Edematous Activity

The anti-edematous effects of free and microencapsulated ibuprofen and escin were evaluated using the carrageenan-induced paw edema model in rats. Edema development was monitored for 24 h after treatment administration by measuring the degree of swelling of the carrageenan-treated hind paw.

**Figure 1:**
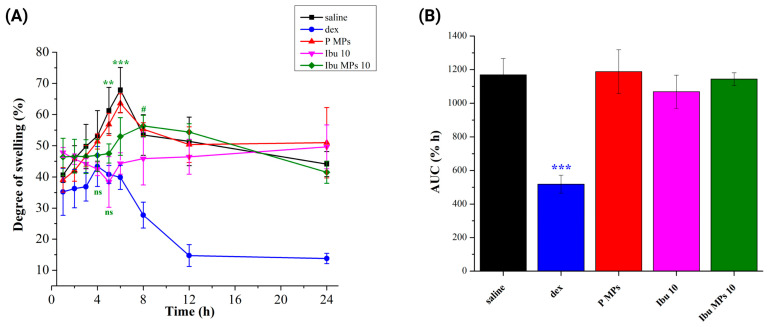
Anti-edematous effect of ibuprofen formulations in the carrageenan-induced paw edema model. (**A**) Time course of paw swelling (%) over 24 h after treatment administration. (**B**) Area under the paw edema–time curve (AUC) calculated from the data shown in panel (**A**). Values are presented as mean ± SD (*n* = 6 per group). The color of the significance markers corresponds to the color of the respective treatment group. **—*p* < 0.01 and ***—*p* < 0.001 versus the negative control group (saline); ns—not significantly different from the dexamethasone-treated group; #—*p* < 0.05 versus the ibuprofen suspension-treated group. To improve figure readability, only selected statistically significant comparisons are shown graphically, while all remaining statistically significant differences are described in the text. Saline—sodium chloride solution for infusion (9 g/L), 1 mL/kg (p.o.); dex—dexamethasone sodium phosphate, 6 mg/kg (i.v.); P MPs—placebo microparticles, 10 mg/kg (p.o.); Ibu 10—ibuprofen suspension, 10 mg/kg (p.o.); Ibu MPs 10—ibuprofen-loaded microparticles, 10 mg/kg (p.o.). p.o.—oral administration; i.v.—intravenous administration.

The effects of the ibuprofen formulations are shown in Figure 1.

Carrageenan administration induced progressive paw edema in the negative control group (saline), with swelling increasing from 40.62 ± 2.41% at 1 h to a maximum of 67.90 ± 7.25% at 6 h, followed by a gradual decline during the later observation period (Figure 1A). Repeated-measures analysis of variance (RM-ANOVA) showed significant effects of time (*p* < 0.001), treatment (*p* < 0.001), and the interaction between time and treatment (*p* < 0.001).

Dexamethasone (positive control) markedly reduced paw swelling compared with the negative control group and resulted in the lowest overall edema response, as indicated by a significantly lower area under the paw edema–time curve (AUC) (518.29 ± 57.13%·h vs. 1168.64 ± 94.55%·h; *p* < 0.001; Figure 1B). Placebo microparticles did not significantly affect paw edema development. Paw swelling values and AUC in the placebo microparticle group did not differ significantly from those of the saline-treated animals (*p* > 0.05).

For ibuprofen formulations, no significant differences from the negative control group were observed during the first 4 h after administration. At 5 h, ibuprofen-loaded microparticles reduced paw swelling to 47.52 ± 3.08%, compared with 61.30 ± 7.43% in the negative control group (*p* < 0.01). A significant reduction was also observed at 6 h (52.99 ± 6.04% vs. 67.90 ± 7.25%, *p* < 0.001). At 5 h, the anti-edematous effect of ibuprofen-loaded microparticles did not differ significantly from that of dexamethasone (*p* > 0.05). At 8 h, paw swelling in the ibuprofen-loaded microparticle group (56.37 ± 3.38%) was significantly higher than in the ibuprofen suspension group (45.90 ± 8.46%, *p* = 0.045). No significant differences from the negative control group were observed thereafter. Analysis of the overall edema response showed no significant differences in AUC between ibuprofen suspension (1068.57 ± 97.85%·h), ibuprofen-loaded microparticles (1143.57 ± 37.88%·h), placebo microparticles (1188.11 ± 128.73%·h), and the negative control group (1168.64 ± 94.55%·h) (*p* > 0.05).

**Figure 2:**
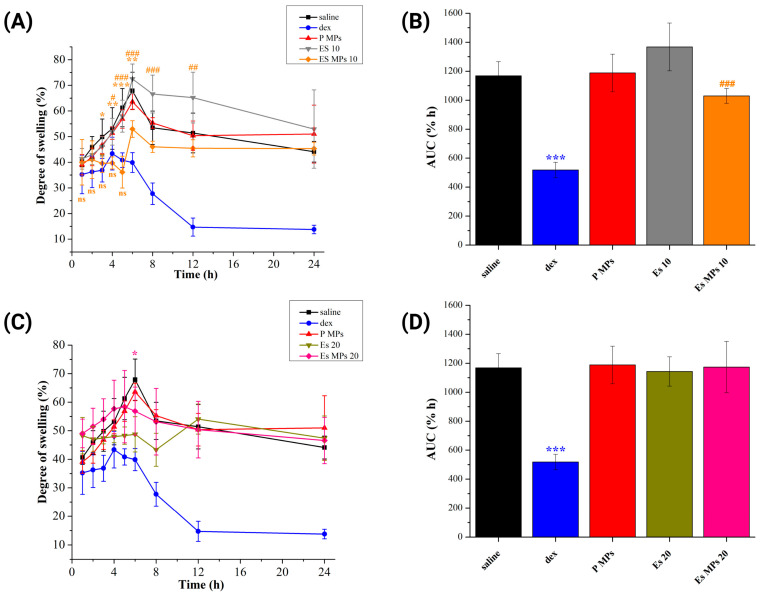
Anti-edematous effect of escin formulations in the carrageenan-induced paw edema model. (**A**) Time course of paw swelling (%) over 24 h after treatment administration in animals receiving escin formulations (10 mg/kg) and the corresponding controls. (**B**) Area under the paw edema–time curve (AUC) calculated from the data shown in panel (**A**). (**C**) Time course of paw swelling (%) over 24 h after treatment administration in animals receiving escin formulations (20 mg/kg) and the corresponding controls. (**D**) Area under the paw edema–time curve (AUC) calculated from the data shown in panel (**C**). Values are presented as mean ± SD (*n* = 6 per group). The color of the significance markers corresponds to the color of the respective treatment group. *—*p* < 0.05, **—*p* < 0.01, and ***—*p* < 0.001 versus the negative control group (saline); ns—not significantly different from the dexamethasone-treated group; #—*p* < 0.05, ##—*p* < 0.01, and ###—*p* < 0.001 versus the escin solution-treated group. To improve figure readability, only selected statistically significant comparisons are shown graphically, while all remaining statistically significant differences are described in the text. Saline—sodium chloride solution for infusion (9 g/L), 1 mL/kg (p.o.); dexamethasone—dexamethasone sodium phosphate, 6 mg/kg (i.v.); P MPs—placebo microparticles, 10 mg/kg (p.o.); Es 10—escin solution, 10 mg/kg (p.o.); Es MPs 10—escin-loaded microparticles, 10 mg/kg (p.o.); Es 20—escin solution, 20 mg/kg (p.o.); Es MPs 20—escin-loaded microparticles, 20 mg/kg (p.o.). p.o.—oral administration; i.v.—intravenous administration.

The anti-edematous effects of escin formulations are shown in Figure 2. RM-ANOVA showed significant effects of time (*p* < 0.001), treatment (*p* < 0.001), and the interaction between time and treatment (*p* < 0.001).

At a dose of 10 mg/kg, escin-loaded microparticles produced the earliest significant reduction in paw swelling. At 3 h, paw swelling was 39.57 ± 3.53% compared with 49.83 ± 7.03% in the negative control group (*p* = 0.012). Significant reductions were also observed at 4 h (39.73 ± 2.35% vs. 53.17 ± 8.10%, *p* = 0.004), 5 h (36.14 ± 6.21% vs. 61.30 ± 7.43%, *p* < 0.001), and 6 h (52.95 ± 3.27% vs. 67.90 ± 7.25%, *p* = 0.001). At all four time points, no significant differences were detected between escin-loaded microparticles and dexamethasone (*p* > 0.05). The corresponding free escin solution (10 mg/kg) did not differ significantly from the negative control group at any time point. Compared with the free escin solution, escin-loaded microparticles produced significantly lower paw swelling at 4 h (39.73 ± 2.35% vs. 43.90 ± 3.41%, *p* = 0.013), 5 h (36.14 ± 6.21% vs. 52.93 ± 4.21%, *p* < 0.001), and 6 h (52.95 ± 3.27% vs. 65.94 ± 5.18%, *p* < 0.001). The AUC of escin-loaded microparticles (1029.43 ± 48.71%·h) was significantly lower than that of the corresponding free escin solution (1367.70 ± 165.74%·h, *p* < 0.001), whereas the AUC of the free escin solution did not differ significantly from the negative control group (*p* > 0.05) (Figure 2B).

At the higher dose of 20 mg/kg, neither free nor microencapsulated escin produced significant reductions in paw swelling compared with the negative control group during the period of maximal edema development. Likewise, no significant differences were observed among the AUC values of free escin (1143.25 ± 85.91%·h), escin-loaded microparticles (1173.29 ± 175.61%·h), placebo microparticles (1188.11 ± 128.73%·h), and the negative control group (1168.64 ± 94.55%·h) (*p* > 0.05) (Figure 2D).

#### 2.1.2. Histological and Immunohistochemical Evaluation of Paw Tissue

**Figure 3:**
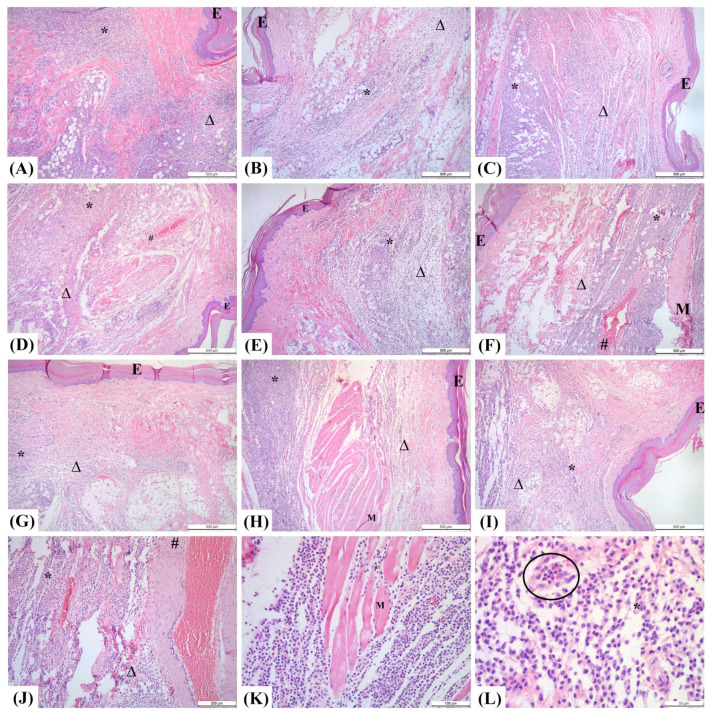
Representative histopathological findings in the carrageenan-induced paw inflammation model 24 h after treatment administration (H&E). (**A**) sodium chloride solution for infusion (9 g/L), 1 mL/kg (p.o.)—negative control group (×5); (**B**) dexamethasone sodium phosphate, 6 mg/kg (i.v.)—positive control group (×5); (**C**) placebo microparticles, 10 mg/kg (p.o.) (×5); (**D**) ibuprofen suspension, 10 mg/kg (p.o.) (×5); (**E**) ibuprofen-loaded microparticles, 10 mg/kg (p.o.) (×5); (**F**) escin solution, 10 mg/kg (p.o.) (×5); (**G**) escin-loaded microparticles, 10 mg/kg (p.o.) (×5); (**H**) escin solution, 20 mg/kg (p.o.) (×5); (**I**) escin-loaded microparticles, 20 mg/kg (p.o.) (×5); (**J**) negative control group showing severe inflammatory changes with inflammatory cell infiltration, edema, and vascular alterations (×10); (**K**) escin solution, 20 mg/kg (p.o.), showing inflammatory infiltrate extending into the underlying muscle tissue (×20); (**L**) negative control group showing neutrophil-predominant inflammatory infiltrate (circled area) (×40); E—epidermis; *—inflammatory infiltrate; Δ—edema; #—vascular alterations; M—muscle tissue damage; p.o.—oral administration; i.v.—intravenous administration.

**Figure 4:**
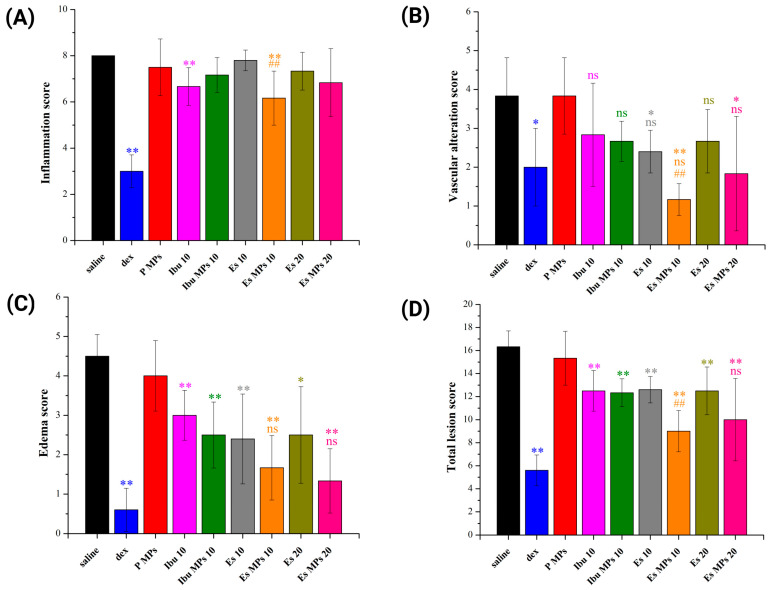
Semiquantitative histopathological scores in paw tissue sections from the experimental groups. (**A**) inflammation score, (**B**) vascular alteration score, (**C**) edema score, (**D**) total lesion score. Values represent mean ± SD (*n* = 6 per group). The color of the significance markers corresponds to the color of the respective treatment group. Levels of statistical significance in comparison with saline: *—*p* < 0.05, **—*p* < 0.01; ns—not significantly different from the dexamethasone-treated group; ##—*p* < 0.01 versus the corresponding non-encapsulated drug formulation. Saline—sodium chloride solution for infusion (9 g/L), 1 mL/kg (p.o.); dex—dexamethasone sodium phosphate, 6 mg/kg (i.v.); P MPs—placebo microparticles, 10 mg/kg (p.o.); Ibu 10—ibuprofen suspension, 10 mg/kg (p.o.); Ibu MPs 10—ibuprofen-loaded microparticles, 10 mg/kg (p.o.); Es 10—escin solution, 10 mg/kg (p.o.); Es MPs 10—escin-loaded microparticles, 10 mg/kg (p.o.); Es 20—escin solution, 20 mg/kg (p.o.); Es MPs 20—escin-loaded microparticles, 20 mg/kg (p.o.). p.o.—oral administration; i.v.—intravenous administration.

Histological examination of paw tissue 24 h after treatment administration was performed using hematoxylin and eosin (H&E) staining to evaluate the degree of inflammatory cell infiltration, interstitial edema, vascular changes, and overall tissue architecture. Representative H&E-stained sections of paw tissue collected 24 h after treatment administration are shown in Figure 3. Figure 4 summarizes the semiquantitative histopathological evaluation of paw tissue 24 h after treatment administration, showing the inflammation score, vascular alteration score, edema score, and total lesion score for all groups.

In the negative control group, extensive infiltration of inflammatory cells, including both mononuclear and polymorphonuclear types, pronounced interstitial edema, and marked vascular alterations were observed (Figure 3A,J,L), resulting in the highest total lesion score among all experimental groups (Figure 4D). The inflammatory infiltrate was distributed throughout the dermis and was especially prominent around dilated and congested blood vessels. In contrast, dexamethasone markedly attenuated inflammatory changes, with only mild inflammatory infiltrate remaining, consisting predominantly of lymphocytes and plasma cells, with only occasional neutrophils (Figure 3B).

Semiquantitative analysis (Figure 4) confirmed significant differences among treatment groups for inflammation score (*p* < 0.001), edema score (*p* < 0.001), vascular alteration score (*p* = 0.002), and total lesion score (*p* < 0.001). Placebo microparticles produced histological findings comparable to those in the negative control group. No significant differences were detected between these groups, indicating that the carrier itself did not contribute to the observed anti-inflammatory effects.

Compared with the negative control group, ibuprofen suspension significantly reduced the inflammation score (6.67 ± 0.84 vs. 8.00 ± 0.00, *p* = 0.007), edema score (3.00 ± 0.61 vs. 4.50 ± 0.55, *p* = 0.006), and total lesion score (12.50 ± 1.76 vs. 16.33 ± 1.37, *p* = 0.006), while the vascular alteration score was not significantly affected (*p* > 0.05). Similar findings were observed for ibuprofen-loaded microparticles, which significantly reduced edema score (2.50 ± 0.84 vs. 4.50 ± 0.55, *p* = 0.003) and total lesion score (12.33 ± 1.21 vs. 16.33 ± 1.37, *p* = 0.003), whereas reductions in inflammation score and vascular alteration score did not reach statistical significance. No statistically significant differences were observed between free and microencapsulated ibuprofen for any of the evaluated histopathological parameters. Compared with dexamethasone, both ibuprofen formulations showed significantly higher inflammation, edema, and total lesion scores, while no significant differences were observed for vascular alteration scores. Histological examination confirmed reduced inflammatory cell infiltration and less pronounced tissue edema in both ibuprofen-treated groups compared with the negative control group (Figure 3D,E).

The most pronounced treatment-related changes among the escin groups were observed for escin-loaded microparticles at 10 mg/kg. This formulation significantly reduced the inflammation score (6.17 ± 1.94; *p* = 0.021), edema score (1.67 ± 0.82; *p* = 0.011), vascular alteration score (1.17 ± 0.41; *p* = 0.020), and total lesion score (9.00 ± 1.79; *p* = 0.004) relative to the negative control group. In addition, the total lesion score was significantly lower than that for free escin at 10 mg/kg (*p* = 0.004), indicating a more pronounced histological improvement following microencapsulation. Consistent with these findings, tissue sections from this group showed reduced inflammatory infiltrates, less edema, and fewer vascular alterations than the corresponding free escin formulation (Figure 3F,G). Moreover, compared with dexamethasone, escin-loaded microparticles at 10 mg/kg showed no statistically significant differences in vascular alteration and edema scores, whereas inflammation and total lesion scores remained significantly higher.

At the higher escin dose (20 mg/kg), both free and microencapsulated formulations reduced edema and total lesion scores relative to the negative control group. However, no statistically significant differences were detected between the free and microencapsulated formulations for any of the evaluated histopathological parameters. Histological findings in these groups were generally comparable (Figure 3H,I).

No statistically significant dose-dependent differences were observed between the 10 and 20 mg/kg escin formulations in the semiquantitative histopathological analysis.

**Figure 5:**
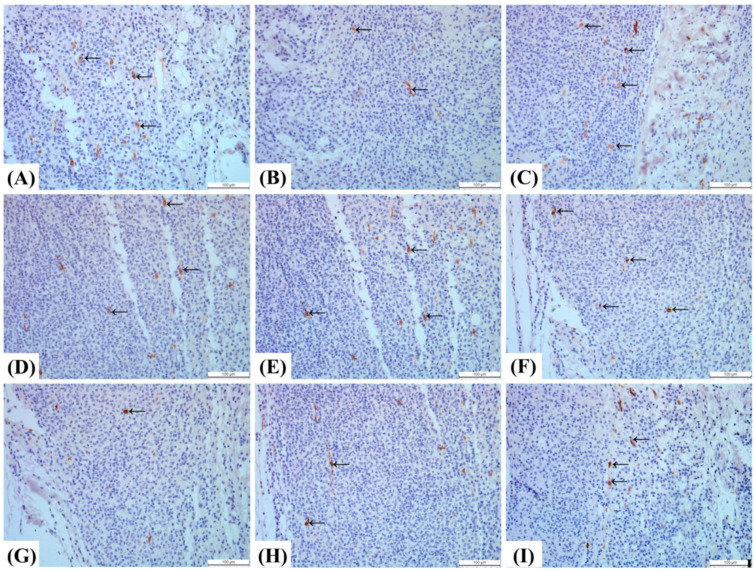
Representative COX-2 immunohistochemical staining in carrageenan-induced paw inflammation model 24 h after treatment administration (×20). (**A**) sodium chloride solution for infusion (9 g/L), 1 mL/kg (p.o.)—negative control group; (**B**) dexamethasone sodium phosphate, 6 mg/kg (i.v.)—positive control group; (**C**) placebo microparticles, 10 mg/kg (p.o.); (**D**) ibuprofen suspension, 10 mg/kg (p.o.); (**E**) ibuprofen-loaded microparticles, 10 mg/kg (p.o.); (**F**) escin solution, 10 mg/kg (p.o.); (**G**) escin-loaded microparticles, 10 mg/kg (p.o.); (**H**) escin solution, 20 mg/kg (p.o.); (**I**) escin-loaded microparticles, 20 mg/kg (p.o.). Arrows indicate representative COX-2-positive cells and areas of positive immunoreactivity. p.o.—oral administration; i.v.—intravenous administration.

**Figure 6:**
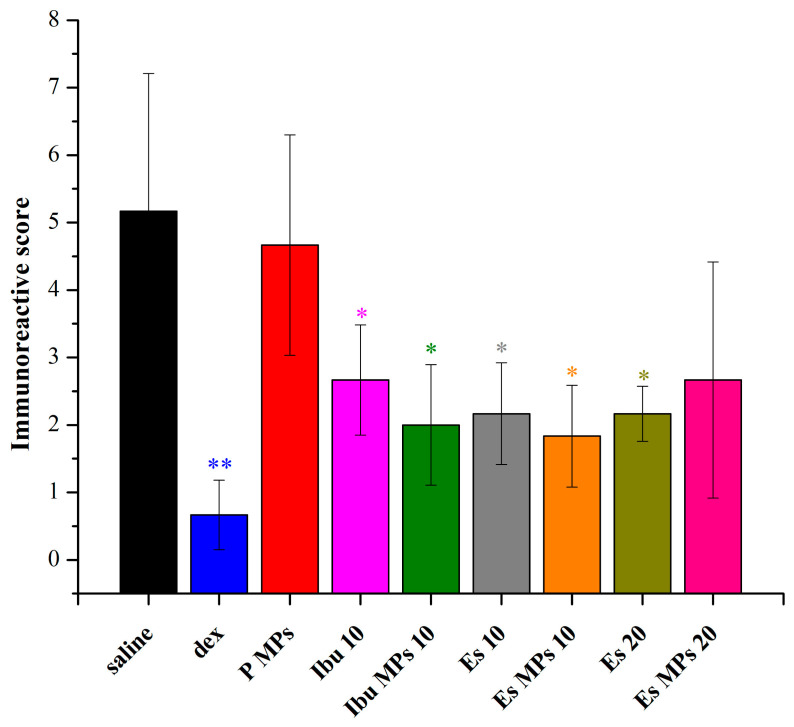
Semiquantitative evaluation of COX-2 immunoreactivity in paw tissue 24 h after treatment administration. Values represent mean ± SD (*n* = 6 per group). The color of the significance markers corresponds to the color of the respective treatment group. Levels of statistical significance compared with saline: *—*p* < 0.05, **—*p* < 0.01. Saline—sodium chloride solution for infusion (9 g/L), 1 mL/kg (p.o.); dex—dexamethasone sodium phosphate, 6 mg/kg (i.v.); P MPs—placebo microparticles, 10 mg/kg (p.o.); Ibu 10—ibuprofen suspension, 10 mg/kg (p.o.); Ibu MPs 10—ibuprofen-loaded microparticles, 10 mg/kg (p.o.); Es 10—escin solution, 10 mg/kg (p.o.); Es MPs 10—escin-loaded microparticles, 10 mg/kg (p.o.); Es 20—escin solution, 20 mg/kg (p.o.); Es MPs 20—escin-loaded microparticles, 20 mg/kg (p.o.). p.o.—oral administration; i.v.—intravenous administration.

COX-2 expression (immunoreactivity) in paw tissue was evaluated immunohistochemically 24 h after treatment administration. Representative COX-2-stained sections are shown in Figure 5, and the corresponding semiquantitative immunoreactive score (IRS) values are presented in Figure 6. COX-2-positive cells were predominantly localized within inflammatory infiltrates and perivascular regions.

Semiquantitative IRS analysis revealed significant differences among treatment groups (*p* < 0.001). The highest COX-2 immunoreactivity was observed in the negative control group (IRS 5.17 ± 2.04), where numerous COX-2-positive cells appeared as individual cells or small clusters, predominantly in the deeper dermis and around blood vessels, corresponding to areas with the most pronounced inflammatory changes (Figure 5A). Similar findings were observed in the placebo microparticle group (IRS 4.67 ± 1.63), with no significant difference compared to the negative control group (*p* > 0.05) (Figure 5C).

Dexamethasone produced the lowest COX-2 immunoreactivity (IRS 0.67 ± 0.52; *p* = 0.006 vs. negative control), with only rare COX-2-positive cells detected within the inflammatory infiltrate (Figure 5B).

Compared to the negative control group, ibuprofen suspension (IRS 2.67 ± 0.82; *p* = 0.044) and ibuprofen-loaded microparticles (IRS 2.00 ± 0.89; *p* = 0.029) significantly reduced COX-2 immunoreactivity. Consistent with these findings, only sparse COX-2-positive cells were observed in the corresponding tissue sections (Figure 5D,E). However, both ibuprofen formulations had higher IRS values than dexamethasone.

Administration of free escin at 10 mg/kg (IRS 2.17 ± 0.75; *p* = 0.036), escin-loaded microparticles at 10 mg/kg (IRS 1.83 ± 0.75; *p* = 0.028), and free escin at 20 mg/kg (IRS 2.17 ± 0.41; *p* = 0.038) also resulted in significantly lower COX-2 immunoreactivity compared to the negative control group. The lowest IRS value among the oral formulations was observed for escin-loaded microparticles at 10 mg/kg. Correspondingly, representative tissue sections from this group showed weaker COX-2 immunostaining than the corresponding free escin formulation (Figure 5F,G). In contrast, escin-loaded microparticles at 20 mg/kg showed a numerically lower IRS value (2.67 ± 1.75) than the negative control group, but the difference was not statistically significant (*p* > 0.05). Only minor differences in COX-2 immunostaining were observed between the free and microencapsulated escin formulations at 20 mg/kg (Figure 5H,I).

Although the mean IRS values generally tended to be lower for microencapsulated formulations than for their corresponding free-drug counterparts, no statistically significant differences were detected between free and microencapsulated ibuprofen or escin formulations. Overall, the immunohistochemical findings were consistent with the histopathological observations, showing reduced inflammatory changes in the active treatment groups compared to the negative control group.

### 2.2. Antinociceptive Activity

**Figure 7:**
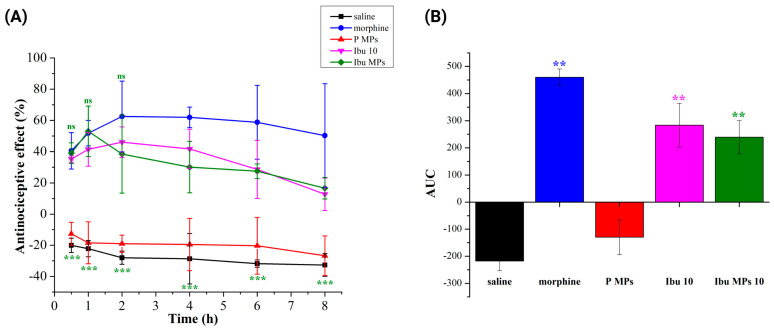
Antinociceptive effect of ibuprofen formulations in the hot plate test. (**A**) Time course of antinociceptive effect (%) over 8 h after treatment administration. (**B**) Area under the antinociceptive effect–time curve (AUC) calculated from the data shown in panel (**A**). Values are presented as mean ± SD (*n* = 6 per group). The color of the significance markers corresponds to the color of the respective treatment group. **—*p* < 0.01 and ***—*p* < 0.001 versus the negative control group (saline); ns—not significantly different from the morphine-treated group. To improve figure readability, only selected statistically significant comparisons are shown graphically, while all remaining statistically significant differences are described in the text. Saline—sodium chloride solution for infusion (9 g/L), 1 mL/kg (p.o.); morphine—morphine sulfate, 5 mg/kg (p.o.); P MPs—placebo microparticles, 10 mg/kg (p.o.); Ibu 10—ibuprofen suspension, 10 mg/kg (p.o.); Ibu MPs 10—ibuprofen-loaded microparticles, 10 mg/kg (p.o.). p.o.—oral administration.

The antinociceptive effects of ibuprofen and escin formulations were evaluated using the hot plate test, which measures centrally mediated nociceptive responses. The results for the ibuprofen formulations are shown in Figure 7.

RM-ANOVA revealed significant effects of time (*p* = 0.004), treatment (*p* < 0.001), and the interaction between time and treatment (*p* = 0.005), indicating that the antinociceptive effect changed over time and differed among the treatments investigated.

The saline-treated group showed a progressive decrease in nociceptive threshold throughout the experiment, whereas morphine, used as the positive control, produced the strongest antinociceptive response, reaching 62.55 ± 22.67% at 2 h and maintaining values above 40% during the entire observation period. Placebo microparticles did not exhibit antinociceptive activity and did not differ significantly from the saline control.

Both ibuprofen formulations significantly increased the nociceptive threshold compared with the saline control. Ibuprofen suspension produced antinociceptive effects ranging from 35.94 ± 1.16% at 30 min to 15.26 ± 9.29% at 8 h, with the maximum response observed at 2 h (48.96 ± 7.62%). Ibuprofen-loaded microparticles showed a more rapid onset of action, reaching their maximum effect at 1 h (53.06 ± 16.16%), followed by a gradual decline to 16.63 ± 6.87% at 8 h. Despite differences in time-effect profiles, no statistically significant differences were detected between free and microencapsulated ibuprofen at individual time points. Analysis of the overall antinociceptive response expressed as AUC also demonstrated significant differences among treatment groups. Both ibuprofen suspension and ibuprofen-loaded microparticles showed significantly higher AUC values than the saline control (*p* = 0.004 and *p* = 0.002, respectively). In contrast, placebo microparticles did not differ significantly from the saline-treated group. No significant difference was observed between the two ibuprofen formulations (*p* > 0.05). Both formulations exhibited lower overall antinociceptive activity than morphine (*p* = 0.008 for ibuprofen suspension and *p* = 0.004 for ibuprofen-loaded microparticles).

**Figure 8:**
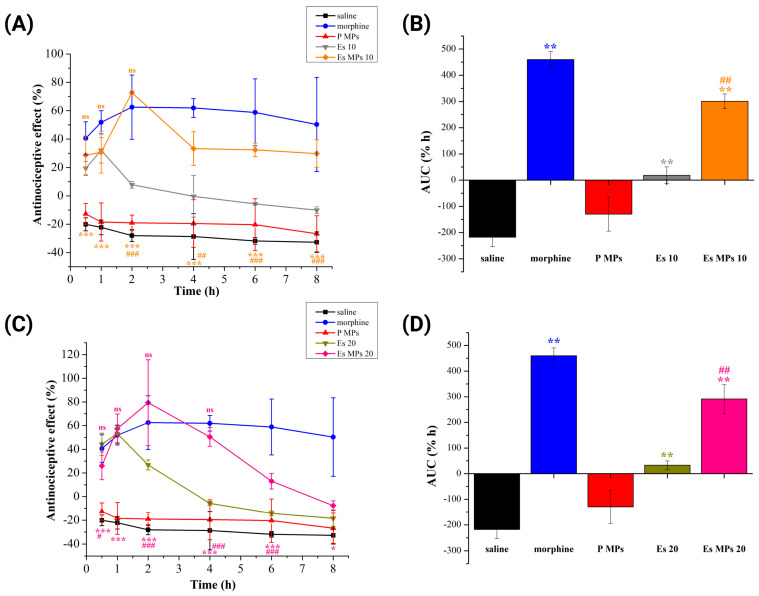
Antinociceptive effect of escin formulations in the hot plate test. (**A**) Time course of antinociceptive effect (%) over 8 h after treatment administration in animals receiving escin formulations (10 mg/kg) and the corresponding controls. (**B**) Area under the antinociceptive effect–time curve (AUC) calculated from the data shown in panel (**A**). (**C**) Time course of antinociceptive effect (%) over 8 h after treatment administration in animals receiving escin formulations (20 mg/kg) and the corresponding controls. (**D**) Area under the antinociceptive effect–time curve (AUC) calculated from the data shown in panel (**C**). Values are presented as mean ± SD (*n* = 6 per group). The color of the significance markers corresponds to the color of the respective treatment group. *—*p* < 0.05, **—*p* < 0.01 and ***—*p* < 0.001 versus the negative control group (saline); ns—not significantly different from the morphine-treated group; #—*p* < 0.05, ##—*p* < 0.01, and ###—*p* < 0.001 versus the escin solution-treated group. To improve figure readability, only selected statistically significant comparisons are shown graphically, while all remaining statistically significant differences are described in the text. Saline—sodium chloride solution for infusion (9 g/L), 1 mL/kg (p.o.); morphine—morphine sulfate, 5 mg/kg (p.o.); P MPs—placebo microparticles, 10 mg/kg (p.o.); Es 10—escin solution, 10 mg/kg (p.o.); Es MPs 10—escin-loaded microparticles, 10 mg/kg (p.o.); Es 20—escin solution, 20 mg/kg (p.o.); Es MPs 20—escin-loaded microparticles, 20 mg/kg (p.o.). p.o.—oral administration.

The antinociceptive effects of escin formulations are shown in Figure 8.

RM-ANOVA showed significant effects of time (*p* < 0.001), treatment (*p* < 0.001), and the interaction between time and treatment (*p* < 0.001), indicating marked differences in both the intensity and duration of the antinociceptive response among treatment groups.

At the 10 mg/kg dose (Figure 8A), escin-loaded microparticles produced the most pronounced and sustained antinociceptive effect. The maximal response occurred at 2 h (72.77 ± 1.40%), exceeding that of morphine (63.25 ± 25.27%), although this difference was not statistically significant. Antinociceptive activity remained evident throughout the observation period, with values of 33.31 ± 11.95%, 32.41 ± 4.72%, and 29.82 ± 9.70% at 4, 6, and 8 h, respectively. In contrast, free escin solution at the same dose produced a less sustained response. After an initial increase in nociceptive threshold during the first 2 h, the effect progressively declined. Escin-loaded microparticles produced significantly greater antinociceptive effects than the corresponding free escin solution at 2, 4, 6, and 8 h (all *p* < 0.001).

At the higher dose of 20 mg/kg (Figure 8C), escin-loaded microparticles also produced a marked antinociceptive response, reaching a maximum of 79.38 ± 36.29% at 2 h. However, the response declined more rapidly than with the 10 mg/kg microparticle formulation, decreasing to 13.02 ± 6.56% at 6 h and becoming negligible at 8 h. Free escin solution at 20 mg/kg produced only a transient response and did not maintain antinociceptive activity beyond the early time points. Escin-loaded microparticles at this dose showed significantly greater effects than the corresponding free escin solution at 2, 4, and 6 h (all *p* < 0.001).

AUC analysis confirmed significant differences among treatment groups (*p* < 0.001). Both escin-loaded microparticle formulations had significantly higher AUC values than the saline control (*p* = 0.002 for both doses), placebo microparticles (*p* < 0.001 for both doses), and the corresponding free escin solutions (10 mg/kg: *p* < 0.001; 20 mg/kg: *p* < 0.001). In contrast, neither free escin formulation differed significantly from the saline control. Comparison of the two escin doses showed no significant differences for either the free escin formulations or the escin-loaded microparticles (*p* > 0.05), indicating comparable overall antinociceptive activity at both dose levels. Morphine produced significantly higher AUC values than all escin formulations (*p* ≤ 0.006).

## 3. Discussion

### 3.1. Anti-Inflammatory Activity

Carrageenan-induced paw edema is one of the most widely used experimental models for evaluating acute inflammation. The inflammatory response develops in a biphasic manner, with the early phase (within the first 2 h) predominantly associated with the release of histamine, serotonin, and bradykinin, while the late phase is characterized by increased production of prostaglandins, cytokines, reactive oxygen species, and infiltration of inflammatory cells, particularly neutrophils. Agents that inhibit prostaglandin synthesis generally exert their most pronounced effects during the second phase of carrageenan-induced inflammation [38]. The time course of the observed anti-edematous effects in this study is consistent with the biphasic nature of carrageenan-induced inflammation. The most pronounced effects of both ibuprofen and escin formulations were observed during the second phase of the inflammatory response (approximately 3–6 h after treatment administration).

As expected, dexamethasone produced the strongest anti-edematous effect, significantly reducing both paw swelling and the overall edema response expressed as AUC. Dexamethasone also markedly reduced inflammation score, edema score, vascular alteration score, total lesion score, and COX-2 immunoreactive score. These findings are consistent with its well-established glucocorticoid-mediated suppression of multiple inflammatory pathways, including inhibition of nuclear factor kappa B (NF-κB) activation and downregulation of pro-inflammatory mediators and enzymes such as COX-2 [39,40,41].

Placebo microparticles did not significantly affect paw swelling, histopathological parameters, or COX-2 expression compared with the negative control group. This indicates that the observed biological effects originated from the incorporated active substances rather than from the chitosan/xanthan gum PEC carrier itself.

For ibuprofen, significant anti-edematous activity was observed primarily during the period of maximal edema development. Ibuprofen-loaded microparticles significantly reduced paw swelling at 5 and 6 h after treatment administration and exhibited anti-edematous effects that did not differ significantly from those of dexamethasone at both time points. It should be noted that dexamethasone was administered intravenously and therefore served as a highly effective positive control with immediate systemic availability. Consequently, the observation that orally administered ibuprofen-loaded microparticles produced anti-edematous effects that were not significantly different from those of dexamethasone at 5 and 6 h is noteworthy despite the different routes of administration. However, the corresponding ibuprofen suspension did not differ significantly from the negative control group at any individual time point. Despite these observations, the overall edema response expressed as AUC did not differ significantly among the ibuprofen suspension, ibuprofen-loaded microparticles, placebo microparticles, and saline-treated groups. These findings indicate that microencapsulation improved the anti-edematous response only during a relatively narrow time window corresponding to the peak inflammatory phase, rather than throughout the entire 24 h observation period.

The limited impact of microencapsulation on the overall anti-inflammatory efficacy of ibuprofen is consistent with the pharmacological properties of this drug. Ibuprofen is a rapidly absorbed NSAID with a relatively short elimination half-life, and its anti-inflammatory activity is primarily mediated through cyclooxygenase inhibition and suppression of prostaglandin synthesis [10]. Previous pharmacokinetic studies by our research group demonstrated increased systemic exposure (higher maximum serum concentration) following encapsulation of ibuprofen in chitosan/xanthan gum PEC microparticles [37]. However, despite these pharmacokinetic improvements, microencapsulation resulted only in a modest enhancement of the anti-inflammatory response, which was evident at selected time points during the peak inflammatory phase.

Histopathological evaluation generally supported the findings from the paw edema model. Both ibuprofen formulations significantly reduced inflammation score, edema score, and total lesion score compared with the negative control group. However, no significant differences were detected between free and microencapsulated ibuprofen for any of the evaluated histopathological parameters. Likewise, both formulations significantly reduced COX-2 immunoreactivity relative to the negative control group, but microencapsulation did not result in a statistically significant additional reduction. Taken together, these findings indicate that although ibuprofen exerted a clear anti-inflammatory effect, the advantage of microencapsulation was modest and was not consistently demonstrated across all evaluated endpoints.

In contrast, a substantially greater benefit of microencapsulation was observed for escin. At a dose of 10 mg/kg, escin-loaded microparticles significantly reduced paw swelling between 3 and 6 h after treatment administration, whereas the corresponding free escin solution did not differ significantly from the negative control group at any time point. Additionally, escin-loaded microparticles produced significantly lower edema values than free escin at 4, 5, and 6 h, and their anti-edematous activity did not differ significantly from dexamethasone during this period. Similar to ibuprofen, the observation that escin-loaded microparticles at 10 mg/kg produced anti-edematous effects not significantly different from those of dexamethasone at several time points is of particular importance, despite oral administration of treatment. The superiority of the microencapsulated formulation was further confirmed by AUC analysis, which showed a significantly lower overall edema response compared with the corresponding free escin solution. These findings are in agreement with our previous pharmacokinetic study, in which escin-loaded microparticles produced significantly greater systemic exposure (higher maximum serum concentration and bioavailability) than the corresponding escin solution [37].

The greater pharmacodynamic benefit observed for escin compared to ibuprofen may also be related to differences in the solid-state properties of the two drugs within the PEC microparticles. Previous physicochemical characterization showed that the amorphous form of escin was preserved after microencapsulation [36], while crystalline ibuprofen underwent only partial amorphization during the microencapsulation process [34]. Because amorphous materials generally exhibit improved dissolution behavior and drug availability [42], retaining the amorphous state of escin may have enabled a more efficient translation of formulation-related improvements into pharmacodynamic effects. Although the contribution of this factor was not specifically investigated in the present study, the observed differences in solid-state properties may have contributed to the distinct pharmacodynamic responses of the two drugs.

Escin exerts anti-inflammatory activity through several complementary mechanisms, including modulation of vascular permeability, inhibition of NF-κB signaling, reduction of pro-inflammatory cytokine production, suppression of phospholipase A2 activity, and downregulation of COX-2 and prostaglandin synthesis [27,28]. Unlike ibuprofen, which primarily exerts its anti-inflammatory activity through cyclooxygenase inhibition and reduced prostaglandin production [10], escin has been reported to affect multiple inflammatory pathways simultaneously. Although these mechanisms were not directly investigated in the present study, differences in the reported mechanisms may contribute to the distinct pharmacodynamic responses observed following microencapsulation of the two drugs.

Among all orally administered formulations, escin-loaded microparticles at 10 mg/kg produced the most pronounced histological improvement. Significant reductions were observed in inflammation score, edema score, vascular alteration score, and total lesion score compared with the negative control group. Furthermore, the total lesion score was significantly lower than that of the corresponding free escin formulation, indicating a clear histological benefit of microencapsulation. Representative tissue sections showed reduced inflammatory infiltrates, less edema, and fewer vascular changes in the escin-loaded microparticle group, corroborating the semiquantitative findings.

The pronounced reduction in vascular alteration score observed for escin-loaded microparticles at 10 mg/kg is consistent with the well-documented venoactive and endothelial-protective properties of escin reported in the literature [27,28]. Since vascular alterations in carrageenan-induced inflammation are closely associated with increased vascular permeability and endothelial dysfunction, this finding is consistent with the proposed role of escin in preserving vascular integrity during acute inflammatory responses [43].

Although all active treatments significantly reduced COX-2 immunoreactivity compared with the negative control group, the lowest IRS values among the orally administered formulations were observed for escin-loaded microparticles at 10 mg/kg. While the differences between free and microencapsulated escin did not reach statistical significance, the reduction in COX-2 immunoreactivity was consistent with the more favorable anti-edematous and histological outcomes observed for this formulation.

The lack of complete agreement between histopathological scores and COX-2 immunoreactivity is not unexpected. While the total lesion score integrates multiple morphological manifestations of inflammation, including edema, vascular alterations, and inflammatory cell infiltration, COX-2 represents a single inducible enzyme involved in prostaglandin biosynthesis rather than the overall inflammatory response [44,45]. Therefore, differences in tissue morphology and lesion severity may not always be fully reflected by changes in COX-2 expression alone.

Interestingly, increasing the escin dose from 10 to 20 mg/kg did not improve anti-inflammatory efficacy. Neither free nor microencapsulated escin at 20 mg/kg significantly reduced paw swelling compared with the negative control group, and no significant differences were detected in AUC values. Similarly, histopathological improvements at this dose were less pronounced than those obtained with escin-loaded microparticles at 10 mg/kg. A possible explanation may be related to altered gastrointestinal transit and absorption dynamics at higher administered doses, particularly considering the larger amount of carrier required for preparation of the higher-dose microparticle formulation [43]. However, this hypothesis requires further investigation.

### 3.2. Antinociceptive Activity

The hot plate test is widely used to evaluate centrally mediated antinociceptive activity because the response involves supraspinal integration of nociceptive stimuli. Although opioid analgesics generally produce the strongest effects in this model, several non-opioid agents, including NSAIDs and compounds with anti-inflammatory activity, may also increase nociceptive thresholds when central pain-processing pathways are affected [46,47,48].

Morphine produced the highest antinociceptive response throughout the experiment and served as an appropriate positive control. In contrast, placebo microparticles did not differ significantly from the saline-treated group, confirming that the carrier system itself did not contribute to the observed antinociceptive activity.

Both ibuprofen formulations significantly increased nociceptive thresholds compared with the negative control group. RM-ANOVA demonstrated significant effects of time, treatment, and their interaction, indicating that the magnitude of the antinociceptive response varied among treatment groups during the observation period. Although ibuprofen-loaded microparticles reached their maximal effect earlier than the suspension formulation, no statistically significant differences were detected between the two formulations at any individual time point or in AUC analysis. Thus, the present data indicate that microencapsulation did not significantly improve the overall antinociceptive efficacy of ibuprofen, despite a tendency toward a faster onset of action.

The antinociceptive activity of ibuprofen is generally attributed to suppression of prostaglandin synthesis in both peripheral tissues and the central nervous system [47,48,49]. The absence of significant differences between free and microencapsulated ibuprofen suggests that the enhanced systemic exposure previously observed after encapsulation [37] did not translate into a measurable improvement in overall analgesic efficacy under the experimental conditions used in this study.

In contrast, escin exhibited markedly different behavior. At both investigated doses, escin-loaded microparticles produced significantly greater antinociceptive effects than the corresponding free escin solutions. At 10 mg/kg, the superiority of the microencapsulated formulation was evident from 2 h onward and persisted throughout the entire observation period. Similar findings were observed at the 20 mg/kg dose, although the duration of the response appeared shorter than that for the lower dose.

The most pronounced antinociceptive response was observed for escin-loaded microparticles at 10 mg/kg, which produced antinociceptive effects that did not differ significantly from those of morphine at several time points and maintained positive antinociceptive activity throughout the 8 h observation period. In contrast, free escin solutions showed substantially weaker and less sustained responses. These observations were confirmed by AUC analysis, where both escin-loaded microparticle formulations showed significantly greater overall antinociceptive activity than the corresponding free escin solutions and placebo microparticles.

The mechanisms responsible for the antinociceptive activity of escin are not fully understood but appear to involve both anti-inflammatory effects and modulation of nociceptive pathways. Previous studies have shown that escin reduces the production of TNF-α, IL-1β, and other inflammatory mediators, suppresses NF-κB activation, and attenuates thermal hyperalgesia in several experimental models [24,50]. The pronounced activity observed in the hot plate test is consistent with previous reports describing the antinociceptive effects of escin in experimental models. The enhanced antinociceptive response observed for escin-loaded microparticles may therefore reflect more efficient pharmacological utilization of escin following microencapsulation.

Interestingly, comparison of the two escin doses revealed no significant differences in overall AUC values for either the free or microencapsulated formulations. Nevertheless, their time-effect profiles differed. Escin-loaded microparticles at 10 mg/kg produced a more sustained response, whereas the 20 mg/kg formulation exhibited a stronger initial effect followed by a more rapid decline. Together with the anti-inflammatory results, these findings suggest that increasing the dose does not necessarily improve pharmacodynamic performance.

These findings further support the superior antinociceptive performance of escin-loaded microparticles relative to the corresponding free escin formulations.

## 4. Materials and Methods

### 4.1. Materials

Medium molecular weight chitosan (190,000–310,000 g mol^−1^; CAS No. 9012-76-4, lot No. STBJ3281), obtained by deacetylation of chitin from crustacean shells and purchased from Sigma-Aldrich (Burlington, MA, USA), was used as the cationic component for the preparation of PECs. The polymer had a degree of deacetylation of 85% and a viscosity of 331 cps (331 mPa·s), determined for a 1% (*w*/*w*) solution in 1% (*w*/*w*) acetic acid. Xanthan gum (CAS No. 11138-66-2; Jungbunzlauer, Basel, Switzerland), pharmaceutical and food grade, served as the anionic component of the PECs and was supplied by Inexall Company d.o.o. (Subotica, Serbia). According to the manufacturer’s specification, the viscosity of xanthan gum, measured as a 1% solution in 1% KCl using a Brookfield LVTD viscometer (Brookfield Engineering Laboratories, Middleboro, MA, USA), spindle 4, 60 rpm, at 25 °C, ranged from 1300 to 1700 mPa·s.

Racemic ibuprofen (CAS No. 51146-57-7; BASF, Ludwigshafen, Germany) and escin free acid (CAS No. 6805-41-0, Indena, Milan, Italy) were used as model drugs. Acetic acid (Ph. Eur.) was used to adjust the pH of the chitosan solution during PEC preparation.

For the in vivo experiments, a 25% urethane solution (Sigma Chemicals Co., St. Louis, MI, USA) was used for general anesthesia prior to paw excision. Carrageenan (Sigma Chemicals Co., St. Louis, MI, USA) served as the inflammatory agent for induction of paw edema in rats. Dexamethasone sodium phosphate injection solution, 4 mg/mL (Dexason^®^, Galenika a.d., Belgrade, Serbia), and oral morphine sulfate solution, 20 mg/mL (Oramorph^®^, L. Molteni & C. dei Fratelli Alitti Società di Esercizio S.p.A., Scandicci, Italy), were used as positive controls in the paw edema and hot plate tests, respectively. Sodium chloride infusion solution, 9 g/L (Natrii chloridi infundibile HF, Hemofarm a.d., Vršac, Serbia), was used as the negative control.

Purified water complying with Ph. Eur. requirements was used throughout all experimental procedures.

### 4.2. Experimental Animals

Adult male Wistar rats weighing 300–350 g and adult male Swiss-Webster mice weighing 25–35 g were used in the study. The animals were obtained from the Military Medical Academy (Belgrade, Serbia) and housed at the Department of Pharmacology and Toxicology, Faculty of Medicine, University of Novi Sad, in Uni-Protect cabinets equipped with a high-efficiency particulate air (HEPA) filtration system (Ehret Labor- und Pharmatechnik GmbH & Co. KG, Emmendingen, Germany).

Animals were housed in transparent polycarbonate cages with wire mesh covers under controlled environmental conditions, including a 12 h light/dark cycle to maintain circadian rhythm, an ambient temperature of 22–25 °C, and relative humidity of 55 ± 1.5%. Standard pelleted diet and water were available ad libitum. Before administration of the tested formulations, food was withdrawn for 12 h and returned 4 h after treatment.

All procedures involving animals were conducted in accordance with the ethical standards for the care and use of laboratory animals defined by EU Directive 2010/63/EU on the protection of animals used for scientific purposes and the Law on Animal Welfare of the Republic of Serbia (Official Gazette of the Republic of Serbia, No. 41/09).

### 4.3. Methods

#### 4.3.1. Preparation of Drug-Free and Drug-Loaded PEC-Based Microparticles

Drug-free (placebo), ibuprofen-loaded, and escin-loaded chitosan/xanthan gum PEC microparticles were prepared using previously optimized formulations and preparation procedures developed by our group. The formulation optimization stage, which included physicochemical characterization and in vitro release studies, was conducted with the same raw material lots and preparation conditions as those later used for the in vivo investigations. Based on the optimization results, one placebo formulation and one drug-loaded formulation for each drug (ibuprofen and escin) were selected for further evaluation. Table 1 summarizes the previously published physicochemical characteristics, in vitro release behavior, pharmacokinetic performance, and safety profile of these optimized formulations to provide the formulation background relevant to interpreting the present pharmacodynamic study.

Aqueous solutions of chitosan and xanthan gum (0.65% *w*/*v* each) were prepared under ambient conditions using a laboratory propeller mixer (RZR 2020, Heidolph Instruments GmbH & Co. KG, Schwabach, Germany). The xanthan gum solution was gradually added to the chitosan solution, which had been previously adjusted to pH 4.6 with acetic acid, and the mixture was stirred for 24 h to allow PEC formation. For drug-loaded systems, ibuprofen or escin was first dispersed in the xanthan gum phase before mixing with the chitosan solution. The final chitosan-to-xanthan gum mass ratio was 1:2, and the drug-to-total polymer ratio was 1:1.

After complex formation, the resulting PEC hydrogels were rinsed with acetate buffer (pH 4.6) to remove unbound components, spread as thin layers in Petri dishes, and dried at room temperature until a constant mass was achieved. The dried films were then ground and sieved (355 μm mesh size) to obtain microparticles.

#### 4.3.2. Preparation of Treatments for In Vivo Studies

Ibuprofen suspension, escin aqueous solution, placebo PEC dispersion, and dispersions of ibuprofen-loaded and escin-loaded microparticles were freshly prepared immediately before administration.

Ibuprofen suspension and escin aqueous solution were prepared by dispersing the appropriate amount of drug in purified water to achieve the required concentrations for each experimental model. PEC dispersions were prepared by dispersing the appropriate amount of microparticles in purified water under magnetic stirring at 400 ± 50 rpm. For the paw edema assay, the final drug concentration was 10 mg/mL, while for the hot plate test, the concentration was adjusted to 2 mg/mL.

All treatments were mixed immediately before administration to ensure homogeneous dosing, particularly for ibuprofen suspensions and microparticle dispersions. Additionally, all microparticle formulations were prepared shortly before the start of the in vivo study to minimize potential stability-related changes.

Ibuprofen was administered as a suspension due to its limited aqueous solubility [10], whereas escin was administered as an aqueous solution [51]. Drug-loaded microparticles were administered as aqueous dispersions because the PEC microparticles readily hydrated and swelled in the aqueous medium [34,35,36]. All reported doses refer to the amount of drug (ibuprofen or escin) rather than the total mass of the microparticle formulation.

#### 4.3.3. Evaluation of Anti-Edematous and Anti-Inflammatory Activity

##### Paw Edema Test

The anti-edematous activity of free and microencapsulated ibuprofen and escin was evaluated using the carrageenan-induced paw edema model in rats. Acute inflammation was induced by intraplantar injection of 0.1 mL of freshly prepared 1% carrageenan solution into the left hind paw. One hour after carrageenan administration, the animals received the respective treatments according to the experimental design. This time point was considered time zero for subsequent measurements.

Fifty-four rats were randomly divided into nine groups (*n* = 6):

- Group 1—sodium chloride 9 g/L solution for infusion, 1 mL/kg (negative control group, saline) (p.o.);
- Group 2—dexamethasone sodium phosphate solution, 6 mg/kg (positive control group, dexamethasone) (i.v.);
- Group 3—placebo microparticles, 10 mg/kg (p.o.);
- Group 4—ibuprofen suspension, 10 mg/kg (p.o.);
- Group 5—ibuprofen-loaded microparticles, 10 mg/kg (p.o.);
- Group 6—escin solution, 10 mg/kg (p.o.);
- Group 7—escin-loaded microparticles, 10 mg/kg (p.o.);
- Group 8—escin solution, 20 mg/kg (p.o.);
- Group 9—escin-loaded microparticles, 20 mg/kg (p.o.).

Paw thickness was measured before carrageenan administration and at predetermined time points after treatment administration (60, 120, 180, 240, 300, 360, 480, 720, and 1440 min) using a calibrated digital caliper (model 202002, Vogel Germany, Kevelaer, Germany). The degree of swelling was calculated according to Equation (1):

Degree of swelling (%) = ((a − b)/b) × 100(1)

where a is the paw thickness at the indicated time point and b is the basal paw thickness.

##### Histological Assessment of Paw Tissue and Immunohistochemistry

After completion of the paw edema study, animals were anesthetized with a 25% urethane solution administered intraperitoneally at a dose of 0.75 g/kg. Once deep anesthesia was confirmed, the carrageenan-treated hind paw was excised for subsequent histological and immunohistochemical evaluation. Animals were euthanized by exsanguination under deep anesthesia in accordance with institutional ethical guidelines.

Soft tissue specimens from the excised paws were fixed in 10% buffered formalin, routinely processed for paraffin embedding, sectioned at 4 μm thickness, and stained with hematoxylin and eosin (H&E).

In addition to standard H&E staining, immunohistochemical analysis was performed on paraffin-embedded sections. Heat-induced antigen retrieval was carried out in citrate buffer (pH 6.0), followed by washing in EnVision™ FLEX Wash Buffer (1:20) (Thermo Fisher Scientific, Waltham, MA, USA). Endogenous peroxidase activity was blocked using EnVision™ FLEX Peroxidase-Blocking Reagent (ready-to-use) (Thermo Fisher Scientific, Waltham, MA, USA). Sections were then incubated with primary antibody against cyclooxygenase-2 (anti-COX-2; 1:500, ab283574, Abcam, Cambridge, UK).

After primary antibody incubation, sections were washed again in EnVision™ FLEX Wash Buffer and incubated for 20 min with an HRP-conjugated secondary antibody using the EnVision™ FLEX detection system (ready-to-use) (Thermo Fisher Scientific, Waltham, MA, USA). Immunoreactivity was visualized using EnVision™ FLEX DAB+ Chromogen (Thermo Fisher Scientific, Waltham, MA, USA), and all sections were counterstained with hematoxylin.

For qualitative analysis of both H&E- and immunohistochemically stained sections, tissue samples were photographed using a Leica DM LB light microscope equipped with a Leica DC100 digital camera (Leica Microsystems, Wetzlar, Germany). Image acquisition and processing were performed using Leica Application Suite software (version 4.12, Leica Biosystems, Deer Park, IL, USA). Descriptive histological evaluation of the sections and semiquantitative assessments were performed independently by two authors blinded to the treatment groups. In cases of disagreement, the final interpretation was established by consensus.

A modified semiquantitative scoring system based on the method described by Hamed et al. [52] was used for histopathological evaluation, performed on 10 high-power fields (magnification ×40). Inflammatory changes were assessed using three parameters: inflammatory cell composition, distribution of inflammatory infiltrates, and infiltrate density. These parameters were summed to generate the inflammation score, allowing separate assessment of different morphological aspects of the inflammatory response. Inflammatory cell composition was classified according to the predominant cellular population within the inflammatory infiltrate. Infiltrate distribution was assessed according to the depth of tissue involvement, whereas infiltrate density was graded according to the abundance of inflammatory cells. The inflammation score was calculated as the sum of inflammatory cell composition, infiltrate distribution, and infiltrate density grades. Edema was assessed in the dermis according to the extent of interstitial fluid accumulation and separation of collagen bundles. Vascular alterations were evaluated based on the severity of vascular dilation, congestion, and vessel wall thickening. Both edema and vascular alteration scores were graded on a scale from 0 to 5, with higher grades indicating more severe tissue alterations. The total lesion score was calculated as the sum of the edema score, vascular alteration score, and inflammation score, with higher values indicating greater overall tissue injury and inflammatory response. Detailed scoring criteria are presented in Supplementary Table S1.

COX-2 expression (immunoreactivity) was evaluated using the immunoreactive score (IRS) system [53], which combines the proportion of positively stained cells and staining intensity, analyzed on 10 high-power fields (magnification ×40). The percentage of positive cells was graded as follows: 0, no positive cells; 1, <10% positive cells; 2, 10–50% positive cells; 3, 51–80% positive cells; and 4, >80% positive cells. Staining intensity was graded as 0 (negative), 1 (weak), 2 (moderate), or 3 (strong). The final IRS was calculated as the product of the percentage score and intensity score, yielding values ranging from 0 to 12, with higher scores indicating greater COX-2 expression.

#### 4.3.4. Evaluation of Antinociceptive Activity

The hot plate test was used to evaluate the antinociceptive effects of free and microencapsulated ibuprofen and escin. Fifty-four Swiss-Webster mice were randomly divided into nine groups (*n* = 6) corresponding to the treatment groups used in the paw edema test. The same group designations were retained, except that morphine sulfate (5 mg/kg, p.o.) was used as the positive control instead of dexamethasone.

Animals were placed individually on a heated metal plate maintained at 50 ± 0.5 °C and covered with a transparent lid. Reaction time was defined as the latency to hind paw licking or shaking and was recorded before treatment and at 30, 60, 120, 240, 360, and 480 min after administration. A cut-off time of 60 s was applied to prevent tissue damage.

The antinociceptive effect was calculated according to Equation (2):

Antinociceptive effect (%) = ((A − B)/B) × 100(2)

where A is the reaction time after treatment and B is the baseline reaction time.

#### 4.3.5. Statistical Analysis

Statistical analysis was performed using IBM SPSS Statistics software (version 21.0, IBM Corp., Armonk, NY, USA). Data are presented as mean ± standard deviation (SD).

The effects of treatment, time, and their interaction in the paw edema and hot plate experiments were analyzed using RM-ANOVA. When the assumption of sphericity was violated, the Greenhouse–Geisser correction was applied. Pairwise comparisons of estimated marginal means were conducted using the Bonferroni adjustment.

To evaluate the overall pharmacodynamic response, AUC was calculated for each animal using the trapezoidal rule. Normality of AUC data was assessed with the Shapiro–Wilk test, and homogeneity of variances was evaluated with Levene’s test. Depending on data distribution and variance homogeneity, AUC values were analyzed either by one-way analysis of variance (ANOVA) followed by Tukey’s HSD post hoc test or by the Kruskal–Wallis test followed by pairwise Mann–Whitney U tests.

Histopathological parameters, including inflammation score, edema score, vascular alteration score, total lesion score, and immunoreactive score, were analyzed using the Kruskal–Wallis test. When significant differences were detected, preplanned pairwise comparisons between treatment groups were performed using the Mann–Whitney U test.

Differences were considered statistically significant at *p* < 0.05.

## 5. Conclusions

Previously optimized chitosan/xanthan gum PEC microparticle formulations loaded with ibuprofen and escin were evaluated for their anti-inflammatory and antinociceptive effects in vivo. Microencapsulation affected the pharmacodynamic performance of the two drugs differently. For ibuprofen, encapsulation improved anti-inflammatory activity only during the peak phase of carrageenan-induced inflammation and produced a slightly earlier antinociceptive response, but did not significantly alter the overall anti-inflammatory or antinociceptive activity compared with the free drug. In contrast, escin-loaded microparticles, particularly at 10 mg/kg, showed significantly greater anti-inflammatory efficacy, as indicated by reduced paw swelling, lower edema AUC, improved histopathological scores, and decreased COX-2 expression. Escin microencapsulation also resulted in stronger and more sustained antinociceptive activity than free escin. These results indicate that the pharmacodynamic benefits of PEC-based microencapsulation are drug-dependent and may extend beyond the improvements in systemic exposure previously observed in pharmacokinetic studies. Overall, chitosan/xanthan gum PEC microparticles appear to be a promising oral delivery platform for escin and may represent a useful strategy for enhancing its therapeutic efficacy in inflammatory conditions.

## Figures and Tables

**Table 1:** Key characteristics of the optimized chitosan/xanthan gum PEC-based formulations selected for pharmacodynamic evaluation *.

Property	Ibuprofen-Loaded Microparticles	Escin-Loaded Microparticles
Drug content (%)	~51.7	~50.3
Entrapment efficiency (%)	~61.5	~64.1
Average particle size (μm)	~15.4	~32.4
Crystallinity	Partial amorphization	Amorphous state retained
In vitro release profile	Prolonged release, zero-order kinetics	Minimal release at acidic pH, enhanced release at intestinal pH
Previous pharmacokinetic findings	Increased serum concentration after oral administration	Improved bioavailability
Safety findings	No significant hepatic, renal, or gastrointestinal toxicity	No significant hepatic, renal, or gastrointestinal toxicity

* Physicochemical characterization and in vitro release data were summarized from previously published studies [34,35,36], while pharmacokinetic and safety findings were adapted from [37]. The table provides a summary of the characteristics of the optimized formulations selected for the present pharmacodynamic evaluation.

## Data Availability

The original contributions presented in the study are included in the article. Further inquiries can be directed to the corresponding author.

## Supplementary Materials

The following supporting information can be downloaded at: https://www.mdpi.com/article/10.3390/md24070225/s1. Table S1. Semiquantitative histopathological scoring system used for evaluation of carrageenan-induced paw inflammation.

## Abbreviations

The following abbreviations are used in this manuscript:

AUC	Area Under the Curve
COX-2	Cyclooxygenase-2
Es	Escin
H&E	Hematoxylin and Eosin
i.v.	Intravenous Administration
Ibu	Ibuprofen
IRS	Immunoreactive Score
MPs	Microparticles
NF-κB	Nuclear Factor kappa B
NSAIDs	Nonsteroidal Anti-Inflammatory Drugs
p.o.	Oral Administration
PEC	Polyelectrolyte Complex
RM-ANOVA	Repeated-Measures Analysis of Variance
