# Supplementary material for: Microparticles Based on Chitosan/Xanthan Gum Polyelectrolyte Complex Modulate the Anti-Inflammatory and Antinociceptive Effects of Ibuprofen and Escin
Source: Mar Drugs. 2026 Jun 26;24(7):225. doi: 10.3390/md24070225 (PMC13413384; doi:10.3390/md24070225)
Supplement: Supplementary file 1 [file marinedrugs-24-00225-s001.zip › marinedrugs-4356845-supplementary.pdf]

Table S1. Semiquantitative histopathological scoring system used for evaluation of carrageenan-induced paw inflammation.

Histopathological parameter	Grades	Description
Edema score	0–5	(0) – None<br>(1) – Minimal interstitial edema<br>(2) – Mild edema with slight separation of collagen bundles<br>(3) – Moderate edema with evident expansion of interstitial spaces<br>(4) – Marked edema with pronounced separation of collagen bundles<br>(5) – Severe diffuse edema with extensive tissue expansion
Vascular alteration score	0–5	(0) – No vascular changes<br>(1) – Minimal vascular dilation<br>(2) – Mild vascular dilation and/or congestion<br>(3) – Moderate vascular dilation and congestion<br>(4) – Marked vascular dilation, congestion, and focal vessel wall thickening<br>(5) – Severe diffuse vascular alterations with prominent congestion and vessel wall thickening
Inflammation score	0–8	
Composition	1–2	(1) – Predominantly lymphocytes and plasma cells<br>(2) – Presence of eosinophils and/or neutrophils in addition to mononuclear inflammatory cells
Distribution	1–3	(1) – Restricted to the superficial dermis<br>(2) – Extending into the deep dermis<br>(3) – Extending between skeletal muscle fibers
Density	0–3	(0) – Absent<br>(1) – Mild<br>(2) – Moderate<br>(3) – Severe

Total lesion score was calculated as the sum of inflammation score, edema score, and vascular alteration score.
